# CDK6 Inhibition: A Novel Approach in AML Management

**DOI:** 10.3390/ijms21072528

**Published:** 2020-04-05

**Authors:** Iris Z. Uras, Veronika Sexl, Karoline Kollmann

**Affiliations:** 1 Department of Pharmacology, Center of Physiology and Pharmacology & Comprehensive Cancer Center (CCC), Medical University of Vienna, 1090 Vienna, Austria; iris.urasjodl@meduniwien.ac.at; 2 Institute of Pharmacology and Toxicology, University of Veterinary Medicine, 1210 Vienna, Austria; veronika.sexl@vetmeduni.ac.at

**Keywords:** AML, CDK6, palbociclib, MLL-AF9, FLT3, RUNX1-ETO, JAK2-V617F

## Abstract

Acute myeloid leukemia (AML) is a complex disease with an aggressive clinical course and high mortality rate. The standard of care for patients has only changed minimally over the past 40 years. However, potentially useful agents have moved from bench to bedside with the potential to revolutionize therapeutic strategies. As such, cell-cycle inhibitors have been discussed as alternative treatment options for AML. In this review, we focus on cyclin-dependent kinase 6 (CDK6) emerging as a key molecule with distinct functions in different subsets of AML. CDK6 exerts its effects in a kinase-dependent and -independent manner which is of clinical significance as current inhibitors only target the enzymatic activity.

## 1. Introduction

Acute myeloid leukemia (AML), which accounts for 75% of acute leukemia, is an aggressive disease with a complex and heterogeneous background characterized by rapid proliferation of hematopoietic progenitor cells frequently lacking terminal differentiation. The treatment modalities for AML include chemotherapy and allogeneic hematopoietic stem cell (HSC) transplantation. Despite these efforts, the outcome is poor with a five-year relative survival of only 17–19% [1,2]. The heterogeneity of the disease poses a huge problem for therapeutic strategies—AML comprises a group of morphologically and genetically distinct malignancies characterized by an aberrant clonal proliferation of myeloid progenitor cells. Hematopoietic stem cells or early myeloid precursors accumulate genetic and epigenetic alterations which lead to clonal expansion and eventually a block of myeloid differentiation [2,3]. The identification of causes and drivers of the pathogenesis of AML enabled the development of small molecules that target the disease on a molecular level. Various compounds including tyrosine kinase inhibitors, immune checkpoint inhibitors, cell-cycle inhibitors, monoclonal or bispecific T-cell engager antibodies, and metabolic and proapoptotic agents are currently under investigation in clinical trials [4,5]. This novel and exciting repertoire of strategies, occasionally in combination with standard chemotherapeutics, has drastically broadened the opportunities of AML treatment. In this review, we focus on the disease contribution and therapeutic targeting of selected cell-cycle kinases in the development and progression of AML. 

## 2. The Specific Functions of CDK6

**Figure 1:**
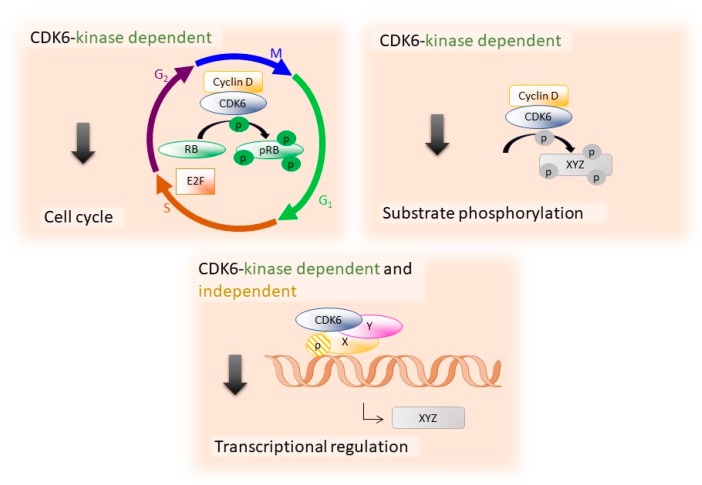
CDK6 promotes cell-cycle progression and phosphorylates various substrates in a kinase-dependent manner and regulates transcription kinase-dependent as well as kinase-independent.

The cyclin-dependent kinase 6 (CDK6) and its close relative CDK4 are critical regulators of cell-cycle progression: in complexes with D-type cyclins, they play redundant roles in relieving retinoblastoma (RB)-mediated transcriptional repression to promote exit from G_1_ to S phase (Figure 1). The simultaneous deletion of both CDKs induces late embryonic lethality in mice due to defects in hematopoiesis [6,7]. Loss of *Cdk6* alone is compatible with life but leads to defects in hematopoietic cell proliferation and mild anemia [7,8]. There is increasing evidence for additional substrates and functional differences between these two kinases that go beyond the control of cell cycle [9] (Figure 1). Contrary to *CDK4*, the *CDK6* gene is frequently amplified or overexpressed in a variety of human lymphomas and leukemias [7,10,11,12,13,14,15,16,17,18,19,20,21]. During the last years, it has been shown that CDK6 but not CDK4 is a direct regulator of transcription in a kinase-dependent and -independent manner, interacting with a range of transcription factors including members of the signal transducer and activator of transcription (STAT) and activator protein 1 (AP-1) family [8,22,23,24,25,26,27]. Besides inducing the transcription of the tumor suppressor p16^INK4A^ as an endogenous feed-back loop, CDK6 also mediates the transcription of vascular endothelial growth factor A (VEGF-A), a well-characterized angiogenic factor and tumor promoter, thereby linking two hallmark cancer features [28,29]. In addition, CDK6 stabilizes the cytoskeletal integrity of erythroid cells on a transcriptional and structural level [8] (Figure 1). 

Recently, CDK6 was assigned a counter-regulatory function during oncogene-induced stress. Throughout transformation, CDK6 is needed to antagonize p53 responses by phosphorylating its DNA binding partners, nuclear transcription factor Y (NFY) and specific protein 1 (SP1), at promoters of p53 antagonizing genes. This finding is reflected in human gene expression signatures from patients with acute lymphoid leukemia (ALL) and myelodysplastic syndrome (MDS). Moreover, an enrichment of p53 negative regulators and NFY target genes showed a positive correlation with CDK6 across myeloid and lymphoid disease entities. These data point at the requirement of additional mutations in the p53 pathway to overcome oncogenic stress when CDK6 kinase activity is blocked. *CDK6*-deficient stable breakpoint cluster region - abelson 1 (BCR-ABL)^+^ leukemic cell lines harbor mutations in the DNA binding domain of p53. The idea that low levels of CDK6 are associated with a higher rate of p53 mutations was verified by analyzing ALL, AML, and MDS patient data. The cohort of patients with low levels of CDK6, due to a 7q deletion which includes the CDK6 gene, have significantly more p53 mutations [30].

Pharmacologic inhibitors of CDK4/6 have recently entered the therapeutic armamentarium of clinical oncologists [31]. Three CDK4/6 kinase inhibitors are currently approved for clinical utility in Europe and the United States: palbociclib (Ibrance, Pfizer), ribociclib (Kisqali, Novartis), and abemaciclib (Verzenios, Lily). All three inhibitors are well-tolerated agents which show similar side effects with some exceptions. Due to bone marrow influence, the main CDK4/6-associated toxicities are neutropenia and leukopenia. Anemia or thrombocytopenia are less frequent. However, due to its greater CDK4 selectivity, abemaciclib demonstrates a lower rate of hematologic adverse events but a higher rate of gastrointestinal toxicities (e.g., grade 3 diarrhea) and fatigue. In the case of ribociclib, QT interval prolongation and hepatobiliary toxicity (increased liver enzymes alanine trans-aminase/aspartate aminase) are notable [32]. In contrast to chemotherapy, CDK4/6-associated toxicities can be limited by dose reductions and dose modifications. 

**Figure 2:**
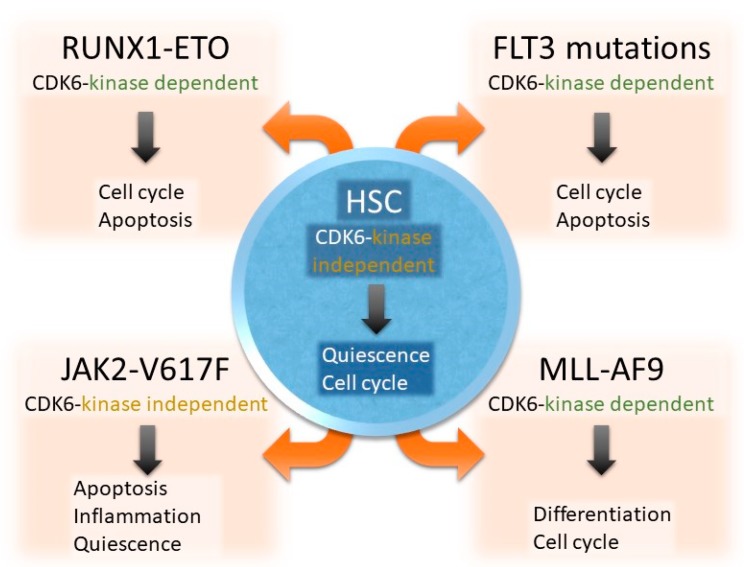
Contribution of CDK6 to pathobiology and treatment of AML. CDK6 regulates cell cycle, apoptosis, stem cell quiescence, differentiation, and inflammation, mainly on a transcriptional level.

The three CDK4/6 inhibitors are used in estrogen receptor (ER)-positive breast cancer based on clinical trials demonstrating improved progression-free survival when combined with antiestrogen therapy, the golden standard for treatment of ER-positive disease [33,34,35]. Abemaciclib is the first one approved as monotherapy [36]. These successes represent only the beginning of the clinical potential of these inhibitors. Clinical data beyond ER-positive breast cancer are currently sparse: single-arm studies have been performed in a variety of cancer types with genomic alterations that are predicted to enhance sensitivity to CDK4/6 inhibition including human epidermal growth factor receptor 2 (HER2)-positive breast cancer, mantle cell lymphoma, liposarcoma, melanoma, non-small cell lung cancer, glioblastoma, neuroblastoma, and malignant rhabdoid tumors [37,38,39,40]. Clinical activity (partial response or prolonged stabilization of disease) has been observed in each study, but in the absence of randomized data, it remains challenging to compare these studies to existing standard care. Although the main mechanism of action is thought to be suppression of RB protein phosphorylation followed by cell-cycle arrest, CDK4/6 kinase inhibitors alter cancer cell biology by means that go well beyond cell-cycle inhibition including modulation of mitogenic kinase signaling, induction of apoptosis, rewiring of transcriptional networks, induction of differentiation, and enhancement of cancer cell immunogenicity that can also be leveraged for therapeutic benefit [26,27,31,41]. In the next sections, we summarize the clinical and biological significance of CDK4/6 kinase inhibition for the treatment of AML (Figure 2).

### 2.1. CDK6 in Hematopoietic Stem Cells

Recently, CDK6 was identified as a key regulator of hematopoietic and leukemic stem cell activation [24,42]. In conditions of hematopoietic stress (e.g., massive blood loss, chemotherapy-induced hematopoietic cell loss or poly (I:C) treatment), quiescent dormant hematopoietic stem cells (HSCs) are rapidly activated to self-renew for replenishing the hematopoietic lineages [43]. Under these stress conditions, dormant HSCs (dHSCs) upregulate CDK6 which exerts kinase-dependent and kinase-independent functions to a low HSC activation. In addition to phosphorylating the RB protein, CDK6 binds the early growth response protein 1 (Egr-1) promoter in a kinase-independent manner and represses its expression. This CDK6-mediated downregulation of Egr-1 is a prerequisite for stem cells to become activated under stress conditions [44] (Figure 1). 

Analogous to “stressed” HSCs, BCR-ABL^p210+^ leukemic stem cells (LSCs) harbor high Egr-1 levels in the absence of CDK6 which hinders them from proliferating and inducing disease. The presence of the BCR-ABL oncogene inflicts “oncogenic stress” in this particular case. Only in the presence of CDK6 or alternatively upon knockdown of Egr-1, BCR-ABL^p210+^ leukemic stem cells form significant numbers of growth factor-independent colonies [24]. A heterozygous EGR-1 deletion is part of 5q deletions, a common recurrent cytogenetic abnormality in AML [45,46]. EGR-1 has been identified as a tumor suppressor as its levels are reduced in several tumor models, including AML [47,48,49]. The upregulation of EGR-1 promotes differentiation in the myeloid lineage [50,51,52,53,54], but its tumor suppressive function depends on the nature of the transforming oncogene [55,56]. 

### 2.2. CDK6 Acts Largely Kinase-Independent in JAK2-V617F^+^ HSCs 

The three main Philadelphia-negative myeloproliferative neoplasms (MPNs)—polycythaemia vera (PV), essential thrombocythaemia (ET), and primary myelofibrosis (PMF)—are characterized by clonal expansion of an early myeloid progenitor cell and overproduction of mature myeloid and erythroid progeny. Clinically, they share the features of bone marrow hypercellularity, increased incidence of thrombosis or hemorrhage, and an increased rate of transformation to acute myeloid leukemia. As these conditions normally precede leukemic transformation, they allow insights into the process of leukemogenesis. 

The first clinical insights into MPN pathogenesis stemmed from the discovery of a single gain-of-function point mutation (Val617Phe) in the nonreceptor tyrosine kinase *JAK2* (janus kinase 2; *JAK2-V617F*) in >95% of patients with PV and in 50% to 60% of patients with ET and PMF [57,58,59,60].

A recent study showed that CDK6 plays a significant role in a JAK2-V617F MPN model. JAK2-V617F mice lacking *CDK6* show a significantly prolonged latency with mitigated clinical symptoms, including increased red blood cell and platelet counts [25]. In line with data from untransformed HSCs [24], CDK6 is needed to release the most dormant JAK2-V617F^+^ HSCs from quiescence which is shown in increased long- and short-term HSC numbers in *JAK2-V617F/Cdk6^-/-^* mice. The underlying mechanism includes an altered cytokine secretion and malignant stem cell activation which is regulated by CDK6 in a largely kinase-independent manner. Moreover, apoptotic players are regulated by CDK6 (e.g., *B-cell translocation gene 2* (*Btg2*) [61], *phorbol-12-myristate-13-acetate-induced protein 1* (*Pmaip1*) [62], *krueppel-like factor 6* (*Klf6*) [63,64], *activating transcription factor 3* (*Atf3*) [61,64,65]) and JAK2-V617F^+^ purified Lineage^−^Sca1^+^cKit+ (LSK) cells lacking *CDK6* show enhanced apoptosis. *CDK6*-deficient LSK cells display altered expression of several negative regulators of nuclear factor kappa-light-chain-enhancer of activated B cells (NF-κB) signaling (e.g., *NF-κB inhibitor zeta* (*NFκBiz*), *suppressor of cytokine signaling 3* (*Socs3*) [66,67,68,69]). These findings implicate CDK6 as a regulator of inflammatory processes which is reflected in diminished Interleukin (IL)-6 and IL-1β levels in the plasma of *JAK2-V617F*/Cdk6^-/-^ mice. Both cytokines influence myeloid lineage differentiation [70,71,72]. Furthermore, several stem cell quiescent genes [44,73,74,75,76,77,78,79,80,81,82,83,84] were found to be dysregulated in the absence of CDK6 under “oncogenic” stress conditions in JAK2-V617F^+^ LSK including *Egr-1* [25]. 

In an attempt to clarify the requirement of CDK6 kinase activity, RNA-Seq experiments have been performed using the CDK4/6 inhibitor palbociclib. These data reveal a predominant kinase-independent role of CDK6 in JAK2-V617F^+^ stem/progenitor cells including the altered apoptosis signaling [25]. Further support for a predominant kinase-independent role of CDK6 in JAK2-V617F^+^ disease stems from studies with human patient samples: primary mononuclear cells from the bone marrow of JAK2-V617F-positive MPN patients treated with palbociclib fail to show increased signs of apoptosis [25]. These data suggest that fine-tuning CDK6 levels may be beneficial for the management of MPN and provides a rationale for the development and implication of CDK6-specific degraders.

## 3. The Role of CDK6 in AML

### 3.1. CDK6 as Driver and Therapeutic Target in MLL Rearrangements 

**Figure 3:**
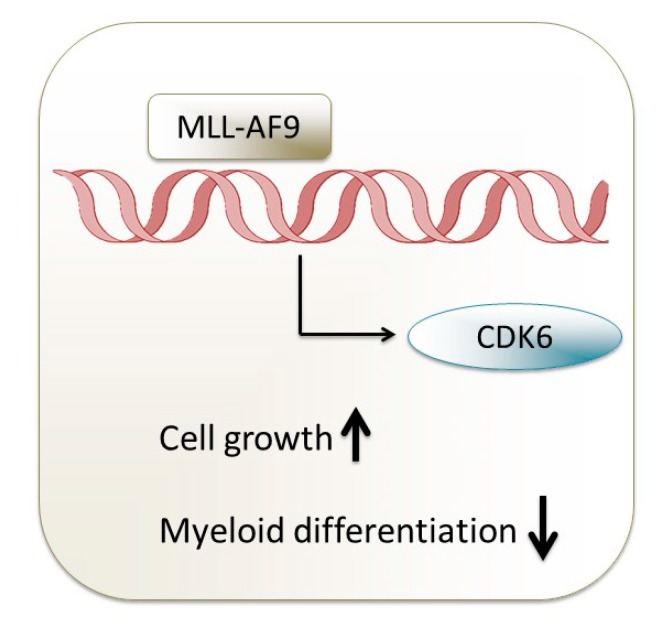
Contribution of CDK6 to pathobiology of MLL-rearranged AML. Once activated by MLL-AF9, CDK6 forces disease progression by blocking myeloid differentiation and inducing cell growth.

The *mixed-lineage leukemia* (*MLL*) gene (now renamed as *Lysine-specific MethylTransferase 2A* (*KMT2A*)) on chromosome 11q23 is disrupted in a particularly aggressive subtype of leukemias. Chromosomal rearrangements of the *MLL* gene occur in ≥80% of infant ALL cases but are less common in older children and adults (5–10%; primarily AML) [85]. A key functional feature of MLL translocations is their ability to lead to aberrant expression of stem cell gene programs and thus to confer leukemia-initiating activity to hematopoietic stem/progenitor cells (HSPCs) [86]. Recently, CDK6 but not CDK4 was found to be a direct target of MLL-fusion proteins in infant MLL-AF4^+^ (MLL-ALL1-fused gene from chromosome 4 protein) ALL [87] and in MLL-AF9^+^ (MLL-ALL1-fused gene from chromosome 9 protein) AML [41]. MLL-AF9 binds the CDK6 locus and its forced expression in wildtype cells elevates levels of CDK6 (Figure 3). It is postulated that CDK6 drives MLL-AF9-mediated disease by inhibiting myeloid differentiation based on the observation that small hairpin RNA (shRNA)-mediated depletion of CDK6 induced myeloid differentiation in MLL-rearranged (MLLr) AML cells. In this system, cell-cycle progression remained unaffected. These effects are specific for CDK6 as rescue experiments with wildtype CDK6 reconstitute myeloid differentiation, a feature not shared by wildtype CDK4. This differentiation phenotype requires the catalytic activity of CDK6 as inhibition of CDK6 by palbociclib mimicked the results obtained with shRNA-mediated knockdown. Palbociclib exposure increases the differentiation of MLLr AML cell lines and mononuclear cells from patient-derived AML cells. In vivo proof of concept for the leukemia-inhibitory and differentiation-inducing role of CDK6 inhibition was provided by a transplantation model of MLL-AF9^+^ AML with shRNA knockdown [41]. An earlier study also reported CDK6 being a target of microRNA29a which regulates myeloid differentiation in HSPCs and AML [88]. These findings have immediate translational implications and paved the way for phase Ib/IIa clinical trials testing palbociclib in patients with MLLr AML (ClinicalTrials.govidentifier: NCT02310243). 

### 3.2. CDK6 Blockage Attacks FLT3-Driven AML via Several Roads 

FMS-like tyrosine kinase 3 (FLT3) is a type III receptor tyrosine kinase that plays an important role in hematopoietic cell survival, proliferation, and differentiation. FLT3 is frequently overexpressed in hematological malignancies and activating mutations are found in AML. FLT3 mutations occur at two distinct hotspots: internal tandem duplications in the juxtamembrane domain (FLT3-ITD) and point mutations, deletions, or insertions in the tyrosine kinase domain (FLT3-TKD), most commonly around D835. TKD mutations drive the activation of the rat sarcoma (RAS), extracellular signal-regulated kinase (ERK), and protein kinase B (PKB/AKT) pathways similarly to ITD mutations which in addition provoke a pronounced activation of STAT5. This difference in STAT5 activation provides an explanation for the differences in disease course, progression pattern, and prognosis observed: while TKD mutations are associated with a milder course of disease, AML patients with ITD mutations develop an aggressive disease and are more prone to relapse. 

FLT3-activated proliferation is caused by enforced expression of D-type cyclins and therefore highly active CDK4/6. Upon treatment with the CDK4/6 kinase inhibitor palbociclib, human FLT3-ITD^+^ AML cell lines display a sustained cell-cycle block. In contrast, the response in FLT3-wildtype cells was transient due to downregulation of p27^Kip^ and reactivation of CDK2. However, this escape mechanism was not observed in human primary patient samples, irrespective of the *FLT3* status [89]. This study suggests activation of CDK2 as a potential resistance mechanism for palbociclib unresponsiveness. It might be worthwhile testing combined inhibition of CDK4/6 and CDK2 which may be limited by its toxicity. 

**Figure 4:**
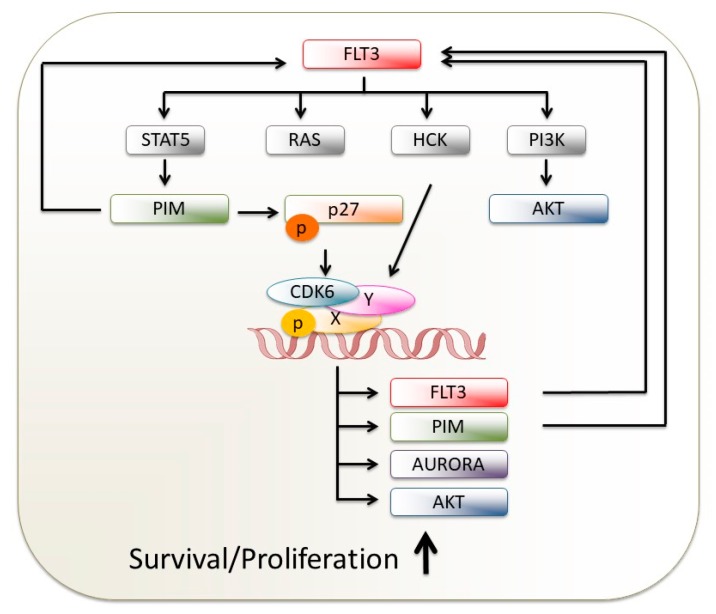
Feed-forward loop in *FLT3*-driven AML. Schematic presentation of signaling pathways initiated by FLT3 mutations and involvement of CDK6 are shown in a simplified fashion.

A novel compound with dual activity against both CDK4/6 and FLT3 has been recently reported [90,91,92]. AMG 925 (FLX925) inhibited AML cell growth in preclinical models and overcame the emergence of resistant clones in FLT3-ITD^+^ cells, a major concern in AML therapy. The treatment also suppressed the proliferation of FLT3 wildtype, RB^+^ AML cells; compared to the single FLT3 kinase inhibition, the clinical response is less dependent on the ITD allele load. Therefore, the AMG 925 inhibitor combining FLT3 and CDK4/6 inhibitory activity is thought to improve clinical performances and leads to a longer lasting response than FLT3 single inhibition. Strikingly, AMG 925 is mostly referred to as a CDK4/FLT3 inhibitor. However, in the light of recent reports, FLT3-ITD-mutated AML cells are more dependent on CDK6 while CDK4 is dispensable [26,27,93]. FLT3-ITD mutation failed to transform primary hematopoietic progenitor cells from *CDK6*-deficient mice, pointing at CDK6 as the prime target of CDK4/6 inhibitors in AML [93]. FLT3-ITD increases CDK6 expression through activation of the v-Src sarcoma (Schmidt-Ruppin A-2) viral oncogene homolog (SRC)-family kinase hemopoietic cell kinase (HCK) (Figure 4); for both kinases approved selective drugs are available for the clinic [33,34,35,36,94]. The combination of compounds targeting the FLT3-HCK-CDK6 axis with classical chemotherapeutics may thus represent a rational strategy for clinical trials in FLT3-ITD^+^ AML. 

A recent study added a further layer of complexity to our understanding. Palbociclib (when applied as monotherapy) induced apoptosis in FLT3-ITD cells; the toxicity is enhanced when combined with FLT3 tyrosine kinase inhibitors (FLT3-TKI) [26]. These effects are specific for FLT3 mutant leukemic cells and were ascribed to the following means: simultaneous application of palbociclib and FLT3-TKI not only blocks cell-cycle progression by dampening phospho-RB (because it suppresses both CDK4/6) [95], but they act synergistically as a result of a dual attack on FLT3 itself. Palbociclib impairs CDK6-mediated transcription of FLT3 while TKI inhibits its activity. The catalytic activity of CDK6 is not required to bind to the FLT3 promoter but is necessary for FLT3 transcription. Downregulation of CDK6 reduced FLT3 levels and diminished downstream signaling, whereas depletion of CDK4 had no effect. CDK6 further facilitates cancer cell survival of FLT3-ITD^+^ AML by directly activating transcription of the proviral integration site for Moloney murine leukemia virus 1 (PIM1), another important leukemogenic driver. This explains the pronounced effects of palbociclib in FLT3-dependent cells when compared to TKI treatment [26]. As PIM kinases phosphorylate and stabilize FLT3 [96], the combined inhibition of CDK6-PIM1-FLT3 interrupts a vicious cycle and feed-forward loop. The same feed-forward loop may explain why leukemic cells with FLT3-ITD alleles have a selective advantage which results in the expansion of FLT3-mutated clones: FLT3 together with PIM1 impairs expression of the CDK inhibitor p27^Kip^ by direct phosphorylation and/or by transcriptional repression; this promotes CDK6 kinase activity which in turn induces the transcription and activity of FLT3 and PIM1 [97,98] (Figure 4). Subsequent work extended these findings in FLT3-D835Y^+^ cells and revealed additional transcriptional targets of CDK6 that are required for the viability and expansion of FLT3-mutant cells. As such, Aurora kinase (AURK) and AKT are identified as CDK6-controlled vulnerabilities (Figure 4). CDK6 binds to chromatin and drives their transcription in a kinase-dependent manner [27]. Although AKT and AURORA kinase inhibitors have significant therapeutic potential in AML, single-agent activity has not been proven overly effective; respective dual inhibitors (FLT3/AURK or FLT3/AKT) are in preclinical development [99,100]. Inhibitors of AURK and AKT signaling, however, effectively synergized with palbociclib in FLT3-ITD and -D835Y-expressing cells. The findings have been confirmed in a FLT3-D835Y^+^ xenograft model and in patient-derived primary biopsies. These data link CDK6 kinase activity to increased apoptosis via the impaired transcriptional regulation of signaling molecules and thus identify CDK6 blockade as a preferable treatment for patients with AML. By attacking multiple kinases including FLT3, PIM1, AURK, and AKT, whose functions are not fully overlapping but all essential for FLT3-ITD- and/or FLT3-TKD-dependent AML growth and survival, CDK6 inhibition may reduce the chances of acquisition of FLT3 resistance mutations and lead to more durable clinical response [26,27]. Strikingly, a cellular model expressing resistance-associated FLT3-ITD-TKD double mutation remained unresponsive when subjected to palbociclib. This discrepancy may be explained by the activation of CDK6-independent alternative downstream signaling and warrants a whole genome-wide in-depth study of differences at gene expression level [27].

### 3.3. CDK6 Kinase Inhibition Targets RUNX1/ETO-Driven Disease Formation

The chromosomal translocation t(8;21) results in the fusion oncogene Runt-related transcription factor 1/myeloid translocation gene on 8 (RUNX1/MTG8 (ETO)) which leads to a specific form of AML. RUNX1/ETO binds to chromatin and regulates the transcription of hundreds of genes and thus ends in the transformation of HSCs. 

A study in RUNX1/ETO human and murine models shows that RUNX1/ETO-driven disease depends on cyclin D2. In these cells, palbociclib induced a proliferation arrest and senescence in vitro and in vivo without provoking myeloid differentiation or quiescence. Palbociclib treatment created a therapeutic vulnerability for KIT inhibition in RUNX1/ETO AML with activation of KIT proto-oncogene mutations [101]. Synergistic effects were also observed upon combining palbociclib with imatinib, an inhibitor of ABL, BCR-ABL, platelet-derived growth factor receptor (PDGFR), and KIT, in human RUNX1/ETO^+^ and KIT-mutated cell lines [102]. 

## 4. CDK6 Protein Degradation Versus CDK6 Kinase Inhibition

The current CDK4/6 kinase inhibitors target the highly conserved ATP-binding pockets of CDKs and inhibit both kinases with similar potency. Recently, specific CDK6 protein degraders have been developed that will target kinase-dependent and -independent functions. These molecules interact with the protein of interest—CDK6—and link it to an E3-ubiquitin ligase resulting in proteosomal degradation. Despite the fact that CDK6 degradation inflicted drastic changes on cellular signaling and transcriptional responses, the human FLT3-ITD^+^ cell line MV4-11 showed growth inhibition without induction of apoptosis [103]. When comparing the CDK6 degrader to palbociclib treatment of MV4-11 cells, no major differences were found in terms of cellular and transcriptional changes [103]. De Dominici et al. showed promising first results using CDK6 degrader in vivo; xenotransplanted mice with patient-derived Philadelphia-positive ALL cells had drastically reduced leukemia burden due to CDK6 degrader treatment [104]. Additional in vitro and in vivo studies are required for the specific AML subtypes respecting all differentiation steps of the leukemic cells to define the consequences of CDK6 protein degradation. 

## 5. CDK6 Inhibitors in Clinical Trials for AML

A phase I clinical trial is ongoing to study potential side effects and optimal dosing of palbociclib when given as monotherapy (during cycle 1) or in combination with either dexamethasone, decitabine, or sorafenib (cycle 2 onward) in advanced AML (ClinicalTrials.govidentifier: NCT03132454). Although well tolerated, palbociclib shows minimal single-agent activity in non-MLL-rearranged refractory/recurrent (R/R) acute leukemia: 50% of patients had progressive disease or died prior to starting cycle 2. Signs of response have been achieved by rational combinations: two AML patients treated with decitabine plus palbociclib had >50% reduction in bone marrow blasts [105]. Pharmacodynamic analysis of patient samples are ongoing. A second single-agent trial enrolls patients with MLL-rearranged leukemia either relapse/refractory or newly diagnosed but ineligible for intensive chemotherapy (AMLSG 23-14 Trial; ClinicalTrials.govidentifier: NCT02310243). The phase Ib of the study has been completed with recruitment of six patients with relapsed/refractory leukemia (AML, *n* = 3; treatment-related AML, *n* = 2; ALL, *n* = 1). Palbociclib shows clinical activity in this prognostically unfavorable subset of leukemia: response assessment revealed one partial remission, three disease stabilizations, and two cases of progressive disease. Four patients completed further treatment cycles (median, 2; range 2–6), with one patient achieving a complete remission with incomplete hematologic recovery after cycle 2. This patient with t(11;19)-positive de novo AML refractory to chemotherapy with daunorubicin and cytarabine relapsed after cycle 6; correlative laboratory studies are underway to determine potential resistance mechanisms [106]. No limiting toxicity was observed during the first 28-day cycle, the limiting toxicity assessment period which took the study forward to the phase IIa expansion part. A phase I dose escalation study of FLX925 (AMG 925), the first dual FLT3 and CDK4/6 inhibitor [90,91,92] in adults with relapsed or treatment refractory AML, demonstrated modest single-agent activity with a dose-limiting toxicity of increased creatinine (ClinicalTrials.govidentifier: NCT02335814). Lower than predicted drug exposure necessitated an increase in dosing frequency to three times a day for appreciable clinical effects but resulted in dose-related adverse effects that limited prolonged exposure [107]. A phase Ib study is underway to assess feasibility and safety of a personalized therapy arm based upon a comprehensive assessment of tumor and patient characteristics (SMMART trial; ClinicalTrials.govidentifier: NCT03878524). Patients with refractory acute myelogenous leukemia will be subjected to palbociclib in combination with another agent from a list of 35 drugs; doses will be escalated on a monthly basis: first month—100% dose Drug A + 25% dose Drug B; second month—100% dose Drug A + 50% dose Drug B; third month—100% dose Drug A + 100% dose Drug B. Another phase I/II trial in adults with AML is designed to evaluate the safety, tolerability, and efficacy of palbociclib in combination with CPX-351 (an U.S. Food and Drug Administration (FDA)-approved nanoscale liposomal coformulation of daunorubicin and cytarabine that allows more effective delivery to the malignant cells) as measured by overall response rate (ClinicalTrials.govidentifier: NCT03844997). Results are not available yet.

## 6. Conclusions

Inhibition of CDK6 is a promising therapeutic strategy in AML, although it remains currently enigmatic which AML subtypes will respond best and how to combine CDK4/6 inhibition. Data so far point towards a major role of CDK6 in different AML subtypes which is not fully understood in terms of transcriptional regulation and substrate activation. Several forms of AML respond with increased apoptosis to CDK6 inhibition. This may be explained by the fact that CDK6 is described as a p53 antagonist during oncogenic stress; CDK6 inhibition enforces the expression of proapoptotic factors which is described during lymphoid leukemia transformation as well as in malignant hematopoietic progenitor cells in a JAK2-V617F model. Therefore, CDK6 inhibition in combination with chemotherapeutics is suggested to be a novel therapeutic strategy by shifting the balance from survival towards apoptosis [30]. Another study confirms that idea, with inhibition of the cell-cycle kinases CDK4/6 by palbociclib prime AML cells for cytotoxic killing by the nucleoside analog cytarabine Ara-C [108]. On the flip side of the coin, low CDK6 levels or inhibition of CDK6 kinase activity may favor the outgrowth of p53-mutated clones. More studies are needed to clarify the time window that allows the acquisition of p53 mutations to make the best use of CDK6 targeting strategies. 

So far, many studies have been performed using human cell lines which might lack the recapitulation of the AML hierarchy and clonal variety of primary AML. In particular, future projects using the novel CDK6 degrader should take this into account. Targeting synergistically vulnerabilities with CDK6 being the common denominator may represent a promising strategy to improve therapy responses and to reduce the incidence of selection of resistance-inducing clones. 

## Abbreviations

AF4	ALL1-fused gene from chromosome 4 protein
AF9	ALL1-fused gene from chromosome 9 protein
ALL	Acute lymphoid leukemia
AML	Acute myeloid leukemia
AP-1	Activator protein 1
Ara-C	Cytarabine
Atf3	Activating transcription factor 3
AURK	Aurora kinase
BCR-ABL	Breakpoint cluster region - abelson 1
Btg2	B-cell translocation gene 2
CDK	Cyclin-dependent kinase
dHSC	Dormant hematopoietic stem cell
EGR-1	Early growth response protein 1
ER	Estrogen receptor
ERK	Extracellular signal-regulated kinase
ET	Essential thrombocythaemia
FDA	U.S. Food and Drug Administration
FLT3	FMS-like tyrosine kinase 3
HCK	Hemopoietic cell kinase
HER2	Human epidermal growth factor receptor 2
HSC	Hematopoietic stem cell
HSPC	Hematopoietic stem/progenitor cell
IL	Interleukin
ITD	Internal tandem duplications
JAK2	Janus kinase 2
Klf6	Krueppel-like factor 6
KMT2A	Lysine-specific MethylTransferase 2A
LSC	Leukemic stem cell
LSK	Purified Lineage^−^ Sca1^+^cKit^+^ cell
MDS	Myelodysplastic syndrome
MLL	Mixed-lineage leukemia
MLLr	MLL-rearranged
MPN	Myeloproliferative neoplasm
MTG8	Myeloid translocation gene on 8
NF-κB	Nuclear factor kappa-light-chain-enhancer of activated B cells
NFY	Nuclear transcription factor Y
NFκBiz	NF-κB inhibitor zeta
OS	Overall survival
PDGFR	Platelet-derived growth factor receptor
PIM1	Proviral integration site for Moloney murine leukemia virus 1
Pmaip1	Phorbol-12-myristate-13-acetate-induced protein 1
PMF	Primary myelofibrosis
Poly (I:C)	Polyinosinic:polycytidylic acid
Protein kinase B	PKB
PV	Polycythaemia vera
R/R	Refractory/recurrent
RAS	Rat sarcoma
RB	Retinoblastoma
RNA-Seq	RNA sequencing
RUNX1	Runt-related transcription factor 1
shRNA	Small hairpin RNA
Socs3	Suppressor of cytokine signaling 3
SP1	Specific protein 1
SRC	v-Src sarcoma (Schmidt-Ruppin A-2) viral oncogene homolog
STAT	Signal transducer and activator of transcription
TKD	Tyrosine kinase domain
TKI	Tyrosine kinase inhibitor
VEGF-A	Vascular endothelial growth factor A
